# Impaired Spatial Reorientation in the 3xTg-AD Mouse Model of Alzheimer’s Disease

**DOI:** 10.1038/s41598-018-37151-z

**Published:** 2019-02-04

**Authors:** Alina C. Stimmell, David Baglietto-Vargas, Shawn C. Moseley, Valérie Lapointe, Lauren M. Thompson, Frank M. LaFerla, Bruce L. McNaughton, Aaron A. Wilber

**Affiliations:** 1 Department of Psychology, Program in Neuroscience, Florida State University, Tallahassee, Florida USA; 2 Neurobiology and Behavior, University of California Irvine, Irvine, California USA; 3 Department of Neuroscience, University of Lethbridge, Lethbridge, Alberta Canada

## Abstract

In early Alzheimer’s disease (AD) spatial navigation is impaired; however, the precise cause of this impairment is unclear. Recent evidence suggests that getting lost is one of the first impairments to emerge in AD. It is possible that getting lost represents a failure to use distal cues to get oriented in space. Therefore, we set out to look for impaired use of distal cues for spatial orientation in a mouse model of amyloidosis (3xTg-AD). To do this, we trained mice to shuttle to the end of a track and back to an enclosed start box to receive a water reward. Then, mice were trained to stop in an unmarked reward zone to receive a brain stimulation reward. The time required to remain in the zone for a reward was increased across training, and the track was positioned in a random start location for each trial. We found that 6-month female, but not 3-month female, 6-month male, or 12-month male, 3xTg-AD mice were impaired. 6-month male and female mice had only intracellular pathology and male mice had less pathology, particularly in the dorsal hippocampus. Thus, AD may cause spatial disorientation as a result of impaired use of landmarks.

## Introduction

Alzheimer’s disease (AD) is a progressive neurodegenerative disorder affecting over 35 million people in the world and is the leading cause of dementia^1–3^. AD involves the formation of amyloid beta (Aβ) plaques, followed by tau pathology in the form of neurofibrillary tangles^2,3^. A critical problem for developing treatments that prevent AD symptomology is that Aβ and tau pathology progress substantially before serious cognitive symptoms appear, leading to a diagnosis long after neurological damage has been done^4^. Individuals with AD have multiple cognitive symptoms including memory and navigational impairments^5,6^. In fact, one of the earliest cognitive symptoms to arise in AD is getting lost^5–7^.

Essentially every mouse model designed to mimic the amyloidosis associated with AD is impaired at spatial navigation^8–12^, for review see^13^. Impaired spatial coding, which could underlie these navigational impairments, has also been reported with less stable spatial maps in 3xTg-AD mice compared to non-transgenic (non-Tg) mice^14^. However, the precise cause of the navigational deficit and impaired spatial coding is unclear. Some studies suggest that navigation is the primary impairment^15–17^, while others suggest the impairments may actually be a consequence of failure to retain information that was learned the previous day (i.e., a memory impairment)^18^, or a combination of memory and navigation impairments^19^. Further, the navigational tasks used to assess impairments assume, but do not explicitly test, that mice are using distal cues to navigate the environment.

Navigational studies in AD have often used tasks designed to engage allocentric (map-like) navigational strategies; however, there is also evidence for egocentric navigational impairments in AD. Specifically, AD can be differentiated from frontotemporal dementia (FTD) based on the presence of an additional egocentric deficit, while allocentric (map-like) impairments are present in both FTD and also AD patients^20^. Consistent with these findings, the parietal cortex (PC), which is thought to play a role in egocentric (e.g., viewer-dependent) navigational strategies^21–27^, is also dysfunctional in mouse models and humans with AD^28^. This dysfunction in PC was attributed to changes in a PC-hippocampal network. Emerging evidence from modeling and experimental studies suggests that this same PC-hippocampal brain network may translate the viewer-dependent (egocentric) representations of landmarks into allocentric (world-centered) coordinates for navigation^21,27,29–33^.

The ability to update knowledge of current location using surrounding landmarks would be critical for navigating, which is dysfunctional in AD^7^. Here, we test the hypothesis that AD may specifically affect the ability to use distal cues to get oriented in space. In order to address this question, we selected a *spatial reorientation task*. This task was previously used to show deficits in aged rats and link this navigational impairment to impaired updating of the hippocampal place field map^34^.

We use a well-characterized triple transgenic mouse model (APPSwe, PS1M146V, and tauP301L) that expresses three major genes associated with familial AD. The 3xTg-AD mouse exhibits plaque and tangle pathologies with a distribution pattern comparable to that observed in humans^35^. In humans with AD, loss of synaptic density precedes neuronal degeneration^36^ and is a better predictor of memory loss than plaques and tangles^37,38^. The 3xTg-AD mouse model also mimics these synaptic changes^39,40^.

## Methods

3xTg-AD male and female mice, and age-matched non-Tg controls, were grouped housed (2–4/cage) in 12:12 h light/dark cycles until the beginning of the experiment. Both 3xTg-AD mice (originally obtained directly from Dr. LaFerla), and non-Tg controls of the same background strain as 3xTg-AD mice, were bred in our vivarium. We assessed male and female mice early in disease progression: 3-month female mice (n = 5/genotype; n = 10), 6-month mice (n = 5/sex/genotype; n = 20), and 12-month male mice (n = 5/genotype; n = 10). Age at beginning of the spatial reorientation task was 3-4, 5–7, or 12–14 months. All experimental procedures were carried out in accordance with the NIH Guide for the Care and Use of Laboratory Animals and approved by the University of California, Irvine and Florida State University Animal Care and Use Committees.

### Pre-training

**Figure 1:**
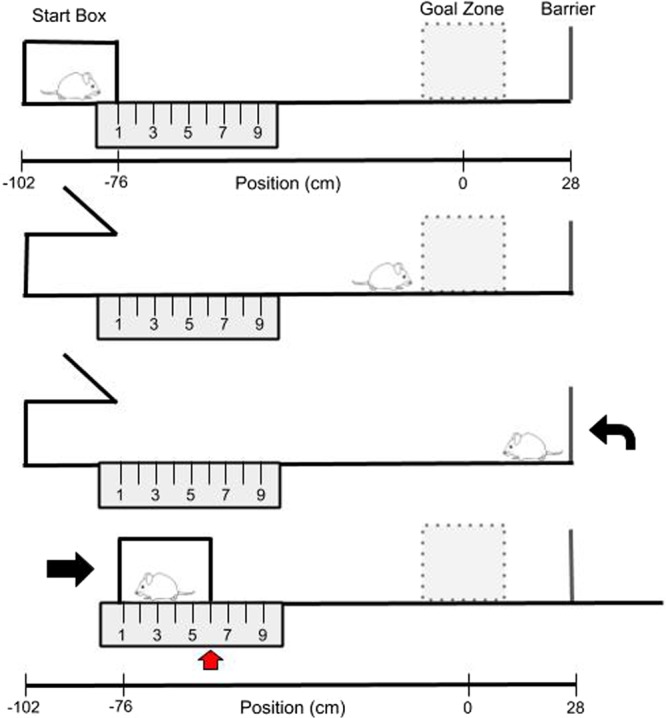
Spatial reorientation task. The goal zone (rewarded location; grey box) is always fixed within the room; however, the start box moves between trials. The sequence of events for each trial are illustrated (*top to bottom*). Each trial ends (*bottom*) with movement of the track to a new randomly selected start location while the mouse is consuming a water reward. Thus, each trial begins with the mouse “lost” with respect to the position of the grey reward zone in the room. After leaving the start box (*second from top*), the mouse gets position estimates initially from self-motion (distance from the start box). As the mouse moves down the track, the position estimation is updated using room-cues. If position is successfully updated using room cues, then the mouse will stop in the reward location for the required delay and obtain a brain stimulation reward. Regardless, if a reward is obtained or not, the mouse shuttles to the end of the track then back to the start box for water reward (*second from bottom*)^29^.

Mice were water deprived to no less than 80% of their beginning body weight and given food *ad libitum*. Mice were then trained to shuttle to a black barrier at the end of a linear track and back for a water reward (*alternation training*). This barrier was positioned so that there was a black background behind it (from the view of the mouse) on the wall. This track was moved to different starting positions, so the length of the track varied (Fig. 1). The starting position was randomly selected from 9 possible start locations for both *alternation training* (spaced over a range of 56–76 cm from the center of the goal zone). Note, all calculations were actually performed in pixel coordinates. The distances listed in cm throughout the paper were estimated for visualization purposes. After reaching criterion (either asymptote, ±6 total runs, 3 out of 4 days, or a total of 50 or more runs down the track and back), the mouse was scheduled for surgery to implant stimulating electrodes, and continued with *alternation training* every other day until the day before surgery when water deprivation ceased.

### Stimulating Electrode Implantation

Once criteria were met on the *alternation training* task, mice underwent surgery to implant bilateral stimulating electrodes targeting the left and right medial forebrain bundle (MFB; 1.9 mm posterior to bregma, 0.8 mm lateral, 4.8 mm below dura). The implantation of these stimulating electrodes allowed for intracranial stimulation of the medial forebrain bundle^41^.

### Stimulation Parameters

Following a 1-week recovery period, mice were placed in a custom 44 × 44 × 44 cm box with a circular nose poke port (Med Associates) mounted on the left half of one wall of the chamber. First, mice were shaped using manual stimulations to approach the nose poke port, then for touching the port, and finally to automatically trigger brain stimulation with beam breaks by nose poking. Following shaping, a custom MATLAB program delivered one 500 ms brain stimulation reward for each beam break in the nose poke port. Over the course of one week, settings were adjusted (171–201 Hz frequency, 50–70 μA current, electrode wire combinations) to achieve maximal response rate. No attempt was made to balance responding across genotype or age; however, responding (nose poke rate) was compared across genotype and age to ensure that differences in reward strength were not likely to contribute to the observed effects. The ideal measure of stimulation effectiveness is use of a running wheel to trigger brain stimulation because operant conditioning is less effective in mice^41^. However, since our task requires mice to stop running for a reward, we did not want to confound the study by training mice to run for brain stimulation then training them on the reverse (stopping for brain stimulation, i.e., reversal learning). Thus, we first assessed brain stimulation responding with an operant task and this worked well for nearly all of the mice in the study. However, a few mice did not have large numbers of beam breaks during operant conditioning (<60 nose pokes) but visually appeared to respond positively to the brain stimulation reward (n = 8 mice; one 3-month 3xTg-AD female, two 3-month non-Tg females, one 6-month 3xTgAD female, one 6-month 3xTg-AD male, one 6-month non-Tg male, and two 12-month 3xTg-AD males). For these low responding mice, we assessed stimulation responsiveness with a task that required alternating for stimulations at either end of the track, *stim alternation*. This was assessed after spatial reorientation training, to prevent a confound of reversal learning. Then, we combined responses for stimulation across these two tasks which produced a continuous distribution with all *stim alternation* values falling below operant training values. In this combined data set we found no significant effects of genotype (3xTg-AD vs non-Tg) on response rate for 3-month females (t_(8)_ = 0.87, *p* = 0.41), for 6-month females (t_(8)_ = 0.10, *p* = 0.92), for 6-month males (t_(8)_ = 0.37, *p* = 0.72), or for 12-month males (t_(8)_ = 0.99, *p* = 0.35). Though unlikely to influence the results given that responding did not differ across genotype, it is possible that higher settings were needed to produce this similar responding in 3xTg-AD mice. Therefore, we also performed the same comparison for the two brain stimulation parameters that we adjusted: frequency and current. There were no significant differences between 6-month 3xTg-AD and non-Tg female mice for frequency (t_(8)_ = 0.65, *p* = 0.54) or current (t_(8)_ = 1.63, *p* = 0.14). Also for 3-month 3xTg-AD and non-Tg female mice, there were no significant differences for frequency (t_(8)_ = 0.63, *p* = 0.54) or for current (t_(8)_ = 1.63, *p* = 0.14). For 6-month males, the list of values for 3xTg-AD and non-Tg mice were identical so t = 0. Also for 12-month 3xTg-AD and non-Tg male mice, there were no significant differences for frequency (t_(8)_ = 0.26, *p* = 0.80) or for current (t_(8)_ = 1.90, *p* = 0.09).

### Spatial Reorientation Training

Once mice were responding maximally, one additional refresher *alternation training* session was conducted, then *spatial reorientation training* commenced adapted from the Rosenzweig *et al*.^34^ task for rats. Throughout the remainder of training and testing, mice shuttled back and forth for a water reward that was delivered in the start box and consumed while the track was moved slowly and smoothly to the next randomly selected starting location (9 possible start locations spaced over a range of 56–76 cm from the center of the goal zone). Random lists were generated using Random.org (random with repeats). Next, an unmarked reward zone was added to the task (28 cm from the barrier at the end of the track) that could automatically trigger a single brain stimulation reward lasting 500 ms. This reward was only delivered if the mouse remained in the zone for a sufficient period before progressing to the end of the track (Fig. 1). This zone was fixed in relationship to the room and cues positioned around the periphery of the room and was marked in camera coordinates. Thus there were no visible markings in the room or on the track indicating the location of the reward zone. Further, since the track was moved to new positions following each trial, any olfactory or other cues on the track could not signal the location of the reward zone. Thus, the reward zone occurred at a variety of physical locations on the track, but always in a fixed location within the room. If the mouse remained in the zone for the duration of a delay period (starting at 0.5 s), then a brain stimulation reward was delivered. The delay was fixed for a given day and was increased by 0.5 s (up to 2.5 s) each time the mouse met a criterion of similar percent correct (±15%), 3 out of 4 days. Following completion of the spatial reorientation task, mice underwent testing on a virtual maze that was similar to the real world spatial reorientation task described above. However, while some animals learned to navigate the virtual maze and complete the virtual version of the task, others were unable to learn how to associate their movements with the virtual maze after a week or more of pre-training. Thus, only a small subset of animals were able to perform this task (n = 2 for 3-month 3xTg-AD females, n = 0 for 3-month non-Tg females, n = 1 for 6-month 3xTg-AD females, n = 2 for 6-month non-Tg females, n = 3 for 6-month 3xTg-AD males, n = 1 for 6-month non-Tg males, n = 4 for 12-month 3xTg-AD males, and n = 3 for 12-month non-Tg males), too few to draw any conclusions, thus the virtual maze data are not reported here. In total, training and testing for both tasks took about 2 months/mouse. As a result, 6-month mice were about 8-months when brain data was acquired.

### Probe Testing

In order to ensure that mice were performing the task as intended, at the conclusion of training and testing, mice underwent two probe tests. First, to ensure that memory for each starting position was not contributing to the observed effects, after reaching criteria for the last 2.5 s delay, mice underwent an additional 2.5 s reward delay session, but with 20 possible random starting positions (same range as the random 9 condition, but with smaller increments between starting locations). Second, after mice reached asymptote on this first probe test (a drop of no more than 15% compared to asymptote from the 2.5 s reward delay), to ascertain that the mice were not using the end barrier as the sole landmark for the reward zone, a second day with 20 random starting positions was administered without the barrier at the end of the track. The same criteria as the first probe test was used for this second probe test. When piloting this behavioral protocol, we found that when the end barrier was not positioned so that it was a black barrier on a black background, performance dropped considerably during this barrier removed probe test. Therefore, care was taken to ensure the black barrier was always positioned with a black background, and a barrier removed probe test was conducted for all data reported here to ensure that no mice were using the barrier as the sole landmark for the reward location. Throughout the spatial reorientation task and probe testing, the mice were video recorded and their position was tracked using video tracking software (Neuralynx; 30 Hz frame rate). Velocity data for each position (each video frame) were extracted from the tracking data and analyzed using custom Matlab (Mathworks) scripts. We converted these velocity values to Z-scores in order to equalize performance across mice with different running speeds.

### Histology

At the conclusion of the experiment, mice were given an intraperitoneal injection of Euthasol and then transcardially perfused with 0.1 *M* phosphate-buffered saline (PBS), followed by 4% paraformaldehyde (PFA) in 0.1 *M* PBS. The whole head was post-fixed for 24 h, to allow for easy identification of the tract representing location of MFB electrodes, and then the brain was removed and post-fixed for another 24 h. Lastly, the brain was cryoprotected in 30% sucrose in 0.1 *M* PBS. Frozen sections were cut coronally with a sliding microtome at a thickness of 40 μm and split into 6 evenly spaced series.

#### 6E10

One series of sections was mounted on slides, incubated in 4% PFA for 4 min, and then rinsed with Tris-buffered saline (TBS). Next, slides were soaked in 70% formic acid for 8–15 min. After rinsing in TBS, slides were incubated in 0.1% Triton-X in TBS for 15 min, followed by 0.1% Triton-X and 2% bovine serum albumin (BSA) in TBS for 30 min. Sections were incubated with anti-β-amyloid 1–16 (mouse, clone 6E10, Biolegend) 1:1000 and anti-NeuN (polyclonal, rabbit, Milipore) in 0.1% Triton-X and 2% BSA in TBS for 2 days. After rinsing with TBS, slides were soaked in 0.1% Triton-X in TBS for 15 min, followed again by 0.1% Triton-X and 2% BSA in TBS for 30 min. Staining was visualized with anti-mouse-alexa-488 (1:1000) and anti-rabbit-alexa-594 (1:500) in 0.1% Triton-X and 2% BSA in TBS for 5–6 hrs. Slides were coverslipped after being rinsed with TBS. Whole slides were imaged as described below, then the coverslip was removed and DAPI (0.01 mg/ml) was added before the slides were re-coverslipped and reimaged. Except for β-Amyloid 1–16 as noted above, histology was performed on free-floating sections. Sections are permeabilized in 0.3% Triton-X and blocked in 3% Goat Serum in TBS, then incubated in primary antibodies.

#### Thioflavin S

A second series incubated in anti-NeuN (1:1000), overnight, was followed by anti-rabbit-alexa-594 (1:500) for 5 h. Sections were rinsed then immersed in a 1% Thioflavin S solution (Sigma) for 9 min, rinsed in dH2O, destained in 70% Ethanol for 5 min, rinsed in dH2O, and then transferred to TBS before mounting onto slides.

#### Phosphorylated tau

A third series was incubated in anti-phosphorylated tau (1:500, monoclonal, mouse, Thermo Scientific) with anti-NeuN (1:1000) overnight. Secondary antibodies were anti-mouse-alexa-488 (1:1000) and anti-rabbit-alexa-594 (1:500) respectively, for 6 h. Sections were rinsed and mounted onto slides.

#### Parvalbumin

Sections were quenched in 0.3% H_2_O_2_ in PBS for 25 minutes, then blocked in 5% goat serum in 0.5% Triton-X TBS for 90 min. Primary antibody mouse anti-parvalbumin antibody (Sigma Aldrich) 1:2000 was added for 2 days followed by a biotinylated goat anti-mouse antibody (Sigma Aldrich) 1:500 for 90 min both in TBS with 0.5% Triton-X. Following this, A and B from the standard Vectastain ABC kit (Vector Laboratories) 1:500 in PBS was added for 1 h. Staining was developed using a DAB (3,3′-Diaminobenzidine tetrahydrochloride hydrate; Sigma Aldrich) solution containing 0.05% DAB and 0.015% H_2_O_2_ in TBS. Sections were rinsed in PBS and mounted onto slides. After air drying, slides were dehydrated in increasing concentrations of alcohol, cleared with Hemo-De and coverslipped with Fisher Chemical Permount™ Mounting Medium.

### Image Acquisition

Whole slides were scanned at 40x magnification using a scanning microscope (NanoZoomer Digital Pathology RS; Hamamatu). In addition, a subset of regions were subjectively examined in greater detail by acquiring image stacks on a confocal microscope (Olympus, FV1000).

### Region of Interest Analyses

**Figure 2:**
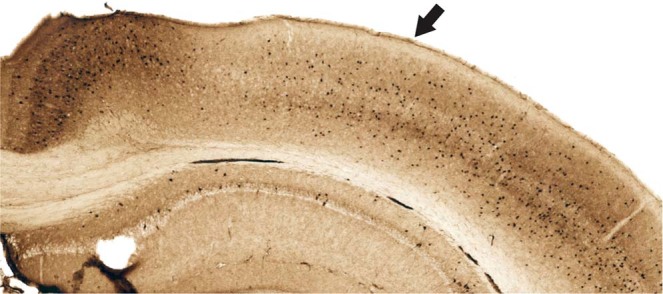
Parvalbumin stained tissue for region of interest analyses. Example of facilitated identification of a region of interest, parietal cortex, using adjacent parvalbumin stained tissue. Parietal cortex (arrow) can be readily identified by the emergence of layer IV (medial boundary) and ending at the lateral border where parvalbumin staining becomes more robust.

The density of cells positive for 6e10 intracellular pathology was estimated for 3 general regions of interest (retrosplenial cortex, CA1 field of the hippocampus, and PC) which were further subdivided as follows. Dorsal CA1 (CA1d) and ventral CA1 (CA1v) were analyzed separately and CA1d was defined as all sections rostral to a point 2.55 mm posterior to bregma. CA1v was considered all sections caudal to that same point. Retrosplenial cortex was subdivided into dorsal (RSCd), ventral (RSCv), and lateral agranular (RSCagl) regions. Next, an outline was manually drawn around each region of interest (ROI) by an experimenter that was blind to group using the manual selection tool in Fiji. ROIs were based on regional boundaries provided by Allen Brain Atlas^42^ and cytoarchitectural differences observed in adjacent series of parvalbumin stained tissue. These boundaries are readily identifiable in NeuN-stained sections, especially when aided with adjacent parvalbumin sections, using standard cytoarchitectural criteria such as cell packing densities and thicknesses of layers. For example, the location of PC within a tissue sample can be more readily identified in a pavalbumin stained section (Fig. 2). The number of 6e10 labeled cells was then calculated for each cortical zone and expressed as the proportion per unit of area. Area calculations were done in pixels and converted to mm for illustrative purposes. These manual counts were acquired for a subset of the sections in the 1:6 series collected for each marker, every other section (i.e., 1:12). Manual counts were performed on 10x equivalent images extracted from 40x scans, ROI contours were drawn on subsampled 2.5x equivalent images.

### Genotyping

We received homozygous 3xTg-AD mice from Dr. Frank LaFerla’s lab. We confirmed that all mice used in the experiment contained each transgene using conventional PCR. DNA was extracted from the tails of each mouse. Homozygosity was confirmed by cutting the PS1 PCR fragment with the *BstEII* restriction enzyme. Only the mutated human PS1 gene contains a *BstEII* cut site and will be cut. The absence of an uncut PCR product indicated that the mouse was indeed homozygous for the human PS1. The presence of overexpressed APP and Tau were also confirmed by PCR. The following primers were used for amplifying the PS1 transgene: 5′-CAC ACG CAA CTC TGA CAT GCA CAG GC-3′ (PS1 Forward) and 5′-AGG CAG GAA GAT CAC GTG TTC AAG TAC-3′ (PS1 Reverse). APP and Tau primers used were 5′-GCT TGC ACC AGT TCT GGA TGG-3′ (APP Forward) and 5′-GAG GTA TTC AGT CAT GTG CT-3′ (APP Reverse) and 5′- GAG GTA TTC AGT CAT GTG CT -3′ (Tau Forward) and 5′-TTC AAA GTT CAC CTG ATA GT-3′ (Tau Reverse) respectively.

### Statistical Analyses

Data were analyzed using two-way repeated measures ANOVAs (genotype x delay, sex x delay, age x delay, and sex x ROI) followed by planned comparisons. Planned comparisons consisted of two-group *F*-tests done within the context of the overall ANOVA (Maxwell and Delaney, 2003), comparing the non-Tg group to the 3xTg-AD group. For all statistical analyses, p < 0.05 was considered significant and the software used for statistical analyses was StatView (SAS Institute Inc.).

## Results

### Female 6-month 3xTg-AD mice are impaired at spatial reorientation

**Figure 3:**
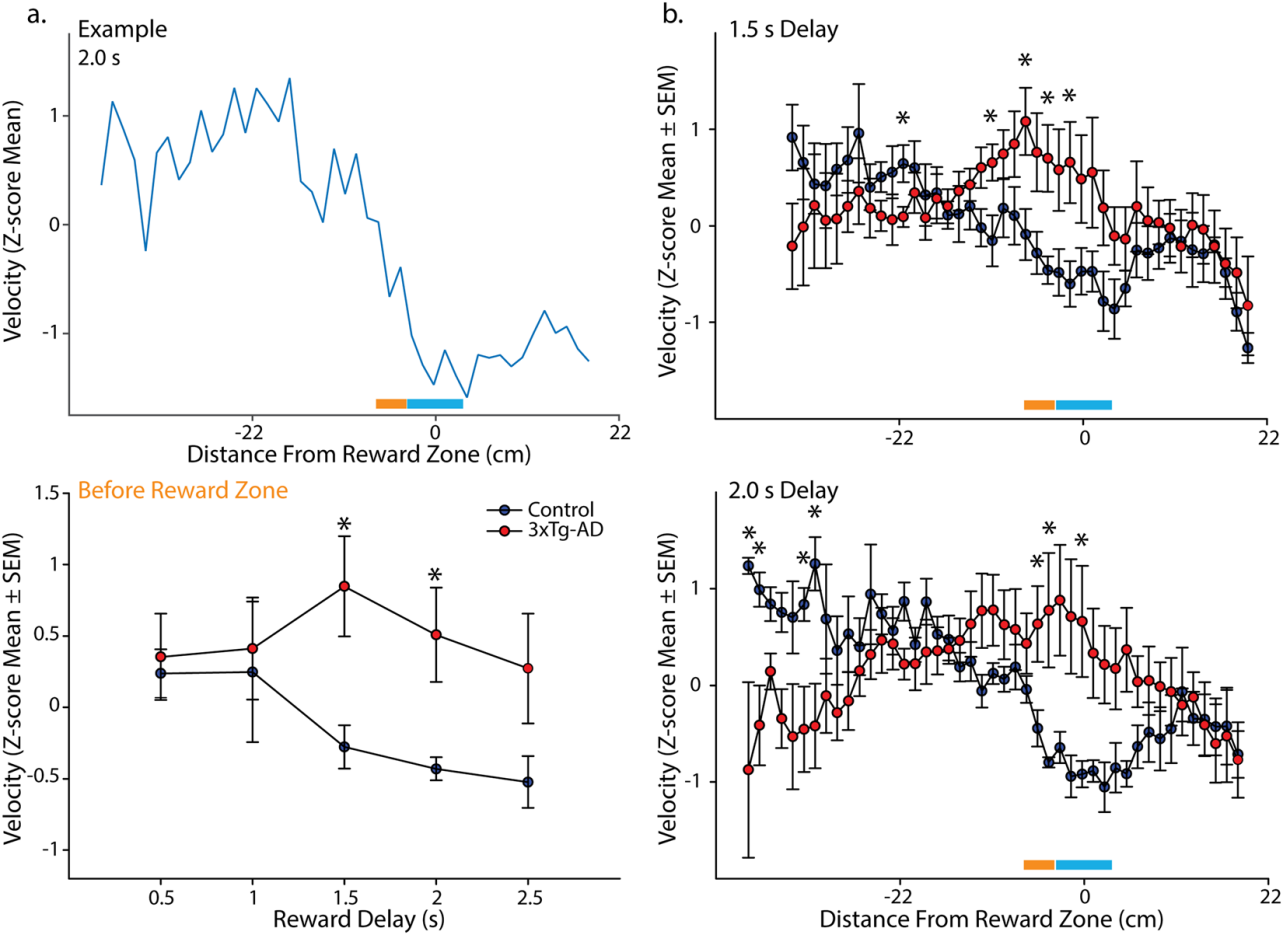
6-month 3xTg-AD female mice are impaired at the spatial reorientation task. (**a**) *Top*. Example mean Z-scored velocity for a single 6-month female non-Tg mouse for the 2.0 s reward delay. This example shows that for a longer reward delay this mouse had learned to slow as it approached the reward zone. *Bottom*. Mean (±SEM) Z-scored velocity from a reward zone radius span of track just prior to the reward zone (orange bar in (**b**); reward zone is the blue bar) for each reward delay (0.5–2.5 s) for 6-month female non-Tg (blue) and 3xTg-AD (red) mice. At more difficult delays, non-Tg mice slowed down more than 3xTg-AD female mice. (**b**) *Top*. Mean (±SEM) Z-scored velocity plotted along distance of the track during the *1*.*5* *s reward delay* for 6-month female non-Tg and 3xTg-AD mice. Non-Tg mice slowed more for the approach and into the reward zone (blue bar) compared to 3xTg-AD mice. *Bottom*. Same as *Top* but for the *2*.*0* *s reward delay*. Again, 3xTg-AD mice failed to slow in the reward zone compared to controls. *p < 0.05.

As mice learn the location of the reward zone, they slow during the approach to the zone, then stop and remain in the zone location for the required delay to obtain a brain stimulation reward (Fig. 3a *top*). Therefore, as a first step in assessing spatial reorientation ability in 3xTg-AD mice, we isolated velocity data (Z-scored) for one reward zone radius just before the front edge of the reward zone (track location used for data shown in Fig. 3a *bottom* is marked on Fig. 3a *top* with an orange bar and the reward zone location is marked on Fig. 3a *top* with a blue bar). This measure allows for comparison of performance across reward delays (i.e., across the range of task difficulty). In addition, measuring the velocity in front of the reward zone avoids contamination from velocity change that results from running slowly through the reward zone at easier delays (e.g., 0.5 s) and stopping after accidentally obtaining the reward. We compared these velocity values during the approach to the reward zone for 3xTg-AD mice to age matched non-Tg controls across reward delays (0.5–2.5 s). Velocity for 6-month female mice did not vary significantly across delay (Fig. 3a *bottom*; F_(4,32)_ = 2.25, *p* = 0.09), or across genotype (F_(1,32)_ = 3.03, *p* = 0.12). However, there was a significant interaction between delay and genotype (F_(4,32)_ = 3.00, *p* < 0.05). We followed up on this significant interaction with planned comparisons assessing the effect of genotype for each delay and found that 3xTg-AD female mice performed significantly worse than non-Tg age matched controls, for the 1.5 and 2 s (Fs_(1,8)_ > 7.60, *p* < 0.05), but not 0.5, 1.0 or 2.5 s (Fs_(1,8)_ < 3.50, *p* > 0.10) reward delays. This suggests that 6-month non-Tg female mice were identifying the location of the reward zone and slowing down in preparation for stopping in the zone, but that 6-month 3xTg-AD females were not.

Next, we examined the mean Z-scored velocities for the full length of the track individually for each reward delay where we observed a significant effect of genotype (the 1.5 and 2 s delays; Fig. 3b). For the *1*.*5* *s delay*, velocity varied across the length of the track (Fig. 3b *top*; F_(41,328)_ = 2.46, *p* < 0.0001), but did not vary across genotype (F_(1,328)_ = 5.01, *p* = 0.06). However, the velocity profile varied significantly as a function of genotype and position in the room (genotype × position interaction: F_(41,328)_ = 1.79, *p* < 0.01). Planned comparisons showed that 6-month female 3xTg-AD mice slowed less than 6-month non-Tg female mice for multiple locations in the room just prior and into the reward zone (−9 cm, −5 cm, −2 cm, 1 cm; Fs_(1,8)_ > 6.17, *p* < 0.05), but not other locations prior to the reward zone (Fs_(1,8)_ < 5.15, *p* > 0.05), nor after the reward zone (Fs_(1,8)_ < 3.09, *p* > 0.12). In addition, non-Tg mice ran faster than 3xTg-AD mice for 1 location near the beginning of the track (−21 cm; F_(1,8)_ = 6.33, *p* < 0.05). Together, this pattern of data suggest that 6-month non-Tg female mice slowed more during the approach and into the reward zone than 6-month 3xTg-AD female mice.

For the 2 *s delay*, there was not a significant main effect of genotype on velocity (F_(1,328)_ = 0.67, *p* = 0.44), but there was a significant effect of position in the room (Fig. 3b *bottom*; F_(44,328)_ = 1.73, *p* < 0.01), and a significant genotype × distance interaction (F_(44,328)_ = 2.58, *p* < 0.0001). Planned comparisons showed that 6-month 3xTg-AD female mice slowed less than non-Tg mice for multiple locations in the room just prior and into the reward zone (−3 cm, −2 cm, 3 cm; Fs_(1,8)_ > 6.17, *p* < 0.05). There were also four locations present towards the beginning of the track where 3xTg-AD female mice were slower than non-Tg female mice (−42 cm, −40 cm, −34 cm, −33 cm; Fs_(1,8)_ > 5.58, *p* < 0.05). No significant difference occurred at other locations prior to the reward zone (Fs_(1,8)_ < 4.97, *p* > 0.06), nor after the reward zone (Fs_(1,8)_ < 2.41, *p* > 0.16). Thus, at both delays (i.e., two second highest difficulty levels), 6-month non-Tg female mice slowed significantly more than 6-month 3xTg-AD female mice whether measures were taken just prior to entering the reward zone or in the reward zone.

#### Probe testing suggests that female mice are using distal cues to get oriented in space

Finally, we used a probe test without the end barrier and our threshold for ‘passing’ this probe test was that a drop in the percentage of trials with successful rewards could be no more than 15%. The reference point for measuring this drop was the asymptote of the 2.5 s reward delay. Similar results were obtained for the random 20 zone list, where again, no more than a 15% drop in correct responding was observed. For both probe tests, with and without the end barrier, mice spent no more than 3 days on each in order to reach the necessary criteria.

#### Impairments in 6-month 3xTg-AD female mice are not a consequence of forgetting from day to day

**Figure 4:**
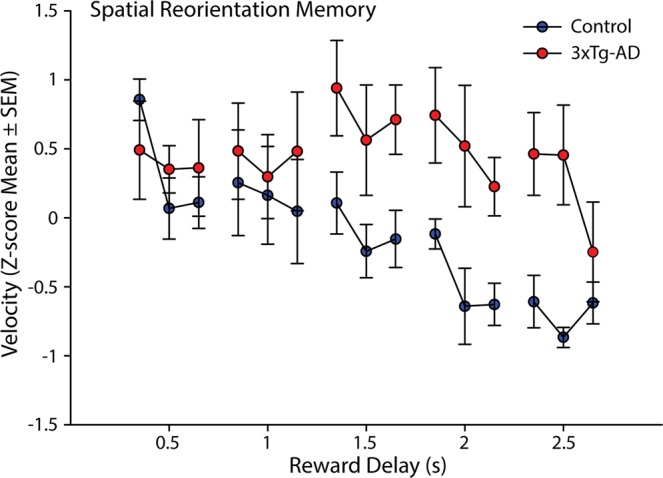
Spatial Reorientation Memory. Z-score mean (±SEM) velocity of the first 1/3, middle 1/3, and the last 1/3 of trials from a reward zone radius span of track just prior to the reward zone for each reward delay (0.5–2.5 s) for 6-month female non-Tg (blue) and 3xTg-AD (red) mice.

Next, to look for evidence that impaired spatial reorientation may be a consequence of forgetting from day to day as reported previously for the Morris water maze^18^, we separated performance for the first 1/3, middle 1/3, and last 1/3 of the trials for each daily session. This was done for the mean Z-scored velocity for the portion of the track just in front of the reward zone at each reward delay as described above (Fig. 4). If the impaired performance in 3xTg-AD mice was a consequence of impaired learning across days, there could be a significant effect observed between the last 1/3 of trials of one delay (e.g. 0.5 s) and the first 1/3 of trials of the following delay (e.g. 1.0 s). Note, this was the comparison used by Billings *et al*.^18^ to show that impaired memory across days produced impaired spatial navigation, replicated as precisely as possible with our data. Visual inspection of the graph reveals that any difference between performance at the end of the preceding day to the beginning of the next day in 3xTg-AD mice is paralleled by a similar shift in non-Tg animals. This pattern is starkly different from that observed by Billings *et al*.^18^, where the within day slope was much greater in transgenic versus non-Tg mice; while in our data, these lines are roughly parallel. This is especially true for the reward delays where significant impairments were observed (1–1.5 s & 1.5–2 s). Similarly, when this is measured statistically as in Billings *et al*.^18^, there were no significant effects observed for either non-Tg (ts_(4)_ < 0.30, *p* > 0.78) or 3xTg-AD mice (ts_(4)_ < 1.85, *p* > 0.14). Thus, while impaired learning and memory could play a role in the deficits we observed here, forgetting across delays was not likely to entirely explain the impaired spatial reorientation we observed in 6-month 3xTg-AD female mice.

### Male 6-month 3xTg-AD mice are not impaired at spatial reorientation

Next, we examined performance of 6-month male mice. As with female mice of the same age (males slightly but not significantly different in the age at first day of spatial reorientation training; t_(8)_ = 0.33, *p* = 0.75), the male mice Z-scored velocity from the portion of the track just in front of the reward zone again did not vary across delay (F_(4,32)_ = 1.12, *p* = 0.37), nor across genotype (F_(1,32)_ = 2.34, *p* = 0.16). However, unlike females of the same age, there was not an interaction between delay and genotype (F_(4,32)_ = 0.52, *p* = 0.72). Thus, 6-month female, but not male, 3xTg-AD mice are impaired at the spatial reorientation task.

#### Differences in markers of pathology but not average velocity corresponds to the sex difference in 6-month 3xTg-AD mice

First, we checked to see if a sex difference in non-Tg male and female mice might contribute to an apparent sex difference in 3xTg-AD mice of the same age. No significant effect of sex on velocity was present (F_(1,32)_ = 3.89, *p* = 0.08), or a significant effect of reward delay (F_(4,32)_ = 1.84, *p* = 0.15). In addition, there was not a significant interaction between sex and delay (F_(4,32)_ = 1.43, *p* = 0.25). Second, to ensure that differences in running speed between the 6-month male and female mice were not contributing to the observed effects, we assessed mean raw velocities. Velocities were only included from the portion of the track where differences in Z-scored velocities were not observed (i.e., from 104 cm to 39 cm in front of the reward zone). We looked at average velocity from the portion of the track where running speed was not expected to vary in order to perform the task (i.e., baseline velocity). Baseline velocity did not vary between sex (F_(1,16)_ = 0.70, *p* = 0.41), or genotype (F_(1,16)_ = 0.30, *p* = 0.59), and there was not a significant interaction between genotype and sex (F_(1,16)_ = 0.56, *p* = 0.46; Mean ± SEM velocity for 6-month non-Tg females: 10.03 ± 1.95; 6-month non-Tg males: 10.16 ± 0.39; 6-month 3xTg-AD females: 9.75 ± 0.87; 6-month 3xTg-AD males: 11.92 ± 1.65).

### Male 6-month 3xTg-AD mice have less pathology than females

**Figure 5:**
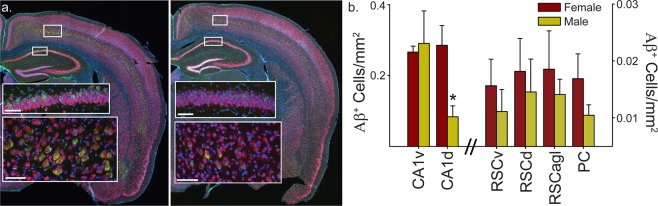
Quantitative analysis shows that 6-month 3xTg-AD female mice have significantly more 6E10 positive neurons in the dorsal hippocampus. (**a**) Sections were triple labeled with DAPI (blue), NeuN (red) and 6E10 (green; Αβ_1-16_ clone). Whole section images were acquired using a slide scanner (40×), then selected regions of interest were imaged using a confocal microscope (insets). Representative staining is shown for a 6-month 3xTg-AD female mouse (*left*) and a 6-month male mouse (*right*). At 6-months, female and male mice have only intraneuronal Αβ, and no extracellular pathology is observed. Representative confocal images are shown in the insets for cortex (*bottom inset*) and CA1 of hippocampus (*top inset*) for each hemi-section. Scale bars are 60 μm. Male mice appear to have less intracellular Αβ in hippocampus and cortex, but what is not captured in these single animal examples is the variability across animals in the same group shown in (**b**). (**b**) Mean (±SEM) density of neurons positive for intraneuronal Αβ_1-16_ for 6-month male and female 3xTg-AD mice. Manual counts of 6E10 (green) positive cells were performed for the dorsal and ventral CA1 field of the hippocampus, dorsal, ventral and lateral agranular retrosplenial cortex and parietal cortex and used to calculate the mean density of 6E10 positive neurons (NeuN^+^) in each ROI for each mouse. The number of 6E10 positive cells was lower in the dorsal hippocampus (CA1d; p < 0.01) of 6-month male (gold) compared to female (maroon) mice. While the general trend showed a lower amount of 6E10 positive cells in 6-month male mice compared to 6-month females in multiple regions, no significant group differences were observed for other regions. Dorsal CA1 (CA1d). Ventral CA1 (CA1v). Dorsal retrosplenial cortex (RSCd). Ventral retrosplenial cortex (RSCv). Lateral agranular retrosplenial cortex (RSCagl). Parietal cortex (PC). *p < 0.01.

When we examined the histology from these 6-month male and female mice (males slightly but not significantly different in the age at perfusion; t_(8)_ = 0.76, *p* = 0.47), it appears that amyloid beta (Aβ) pathology, as measured by the density of the subjective number of 6E10 (Αβ1–16 specific antibody) positive cells, in 6-month female 3xTg-AD mice is greater than in male mice (Fig. 5a). The level of other markers of pathology were very low at this age (thioflavin S, phosphorylated tau; i.e., floor effect) and thus were not compared between males and females (not shown).

Finally, for 6-month male and female 3xTg-AD mice, we quantified this subjective difference in pathology for the CA1 field of the hippocampus (that was previously linked to impaired spatial orientation in aged mice)^34^ and two cortical regions that emerging evidence suggests may be critical for this navigational impairment: the PC and Retrosplenial Cortex (RSC)^27,43^. In addition, given that dorsal hippocampus is thought to be especially critical for spatial navigation, we analyzed dorsal and ventral hippocampus separately^44–47^. We found a significant effect of region (Fig. 5b; CA1d, CA1v, PC, RSCd & RSCv; F_(5,40)_ = 39.26, p < 0.0001) and a significant sex × brain region interaction (F_(5,40)_ = 4.91, p < 0.01), but not a significant effect of sex (F_(1,40)_ = 1.98, p = 0.20). Planned comparisons indicated that female mice had significantly more pathology than male mice in dorsal CA1 (F_(1,8)_ = 12.57, p < 0.01) but no other region (CA1v, PC, RSCd, RSCv & RSCagl; Fs_(1,8)_ < 2.31, ps > 0.17). The general trend indicated that the mean number of positive cells in female mice was higher than in male mice in most areas quantified, but variability across animals was sufficient to prevent a significant difference in the means. This suggests that a sex difference in pathology, but not sex differences in non-Tg animals or differences in running speed, may correspond to the sex difference in impaired spatial reorientation in 6-month 3xTg-AD mice.

### Female 3-month 3xTg-AD mice are not impaired at spatial reorientation

Since impairment in spatial reorientation appeared in female 3xTg-AD mice at 6-months, we assessed 3-month females on the spatial reorientation task to determine if this impairment occurred earlier on in disease progression. Mean Z-scored velocity just in front of the reward zone did not vary across delay (F_(4,32)_ = 0.68, *p* = 0.61) or genotype (F_(1,32)_ = 0.43, *p* = 0.53) and there was not a significant interaction between delay and genotype (F_(4,32)_ = 0.98, *p* = 0.43). Next, we examined the possibility that a developmental difference in the ability of non-Tg mice to perform the task might contribute to the lack of impairment in 3-month 3xTg-AD female mice. That is, the lack of impairment observed in 3-month mice might actually be an inability to perform the task. However, when 3-month and 6-month non-Tg female mice were compared, velocity did not vary with age (F_(1,32)_ = 0.22, *p* = 0.65), or reward delay (F_(4,32)_ = 1.75, *p* = 0.16), and there was not a significant interaction between age and delay (F_(4,32)_ = 0.79, *p* = 0.54). This suggests that performance of 3-month non-Tg mice was not significantly worse than 6-month non-Tg mice and thus provides further evidence that 3-month 3xTg-AD female mice do not have impaired spatial reorientation. Next, to ensure that developmental differences in running speed were not contributing to the observed effects, we assessed mean raw velocities between 3- and 6-month female mice. Velocity did not vary across age (F_(1,16)_ = 0.04, *p* = 0.85), or across genotype (F_(1,16)_ = 0.18, *p* = 0.68) and there was not a significant interaction between genotype and age (F_(1,16)_ = 0.45, *p* = 0.51; Mean ± SEM velocity for non-Tg 3-month females: 9.08 ± 0.26; 3-month 3xTg-AD females: 10.30 ± 0.61; 6-month non-Tg and 3xTg-AD female values are above).

### Male 12-month 3xTg-AD mice are not impaired at spatial reorientation

Since no impairment in spatial reorientation appeared in male 3xTg-AD mice at 6-months, we assessed 12-month males on the spatial reorientation task to determine if an impairment occurred later on in disease progression. Mean Z-scored velocity just in front of the reward zone did not vary across delay (F_(4,32)_ = 1.67, *p* = 0.18) or genotype (F_(1,32)_ = 0.04, *p* = 0.85) and there was not a significant interaction between delay and genotype (F_(4,32)_ = 2.04, *p* = 0.11). Next, to ensure that age-related differences in running speed were not contributing to the observed effects, we assessed mean raw velocities between 6- and 12-month male mice. Velocity did not vary across age (F_(1,16)_ = 0.55, *p* = 0.45), or across genotype (F_(1,16)_ = 0.21, *p* = 0.65) and there was not a significant interaction between genotype and age (F_(1,16)_ = 0.83, *p* = 0.84; Mean ± SEM velocity for non-Tg 12-month males: 12.28 ± 0.78; 12-month 3xTg-AD males: 11.70 ± 1.76; 6-month non-Tg and 3xTg-AD male values are above).

## Discussion

We found that, compared to non-Tg mice, 6-month female 3xTg-AD mice were impaired at spatial reorientation when challenged with a task requiring them use of distal cues to become reoriented. In contrast, male mice (6-months and 12-months) and younger female mice (3-months) were not impaired at spatial reorientation. The impaired spatial reorientation in female mice was observed early in disease progression, when intracellular accumulation of Aβ was apparent, but prior to the appearance of extracellular pathology. The lack of impairments in male mice of the same age might correspond to a difference in the disease progression since the intracellular pathology was significantly reduced in dorsal CA1, compared to 6-month female mice. Together, our data suggest that impairments in spatial reorientation emerge very early in disease progression and that the ability to get reoriented in space may be sensitive to subtle differences in disease progression in early AD.

The impaired spatial reorientation we observed in 3xTg-AD mice is consistent with impairments in spatial learning and memory dependent tasks such as the Morris water maze, Barnes maze, and T-maze in 3xTg-AD mice^15–18,48–50^. Interestingly, the deficit in the Morris water maze was also shown to emerge early in disease progression, before extracellular pathology was apparent; however, the spatial navigation impairment in the water maze appeared to be driven, at least largely, by forgetting from day to day with apparently normal learning across the sessions within a day^18^. It is likely that the impairments we observed are in part a consequence of impaired learning and memory; however, we did not see evidence in our data that impaired forgetting from day to day is completely responsible for the impaired spatial reorientation we observed. This suggests that the task we used may also be sensitive to navigation deficits, presumably related to the emphasis on using distal cues to get reoriented.

Previous studies showing impaired spatial navigation in mouse models of AD have assumed that mice were using distal cues to navigate; however, these tasks do not require mice to repeatedly make use of distal cues to get oriented in space, thus potentially failing to fully engage this process^16–18,48–51^. Further, rats can use proximal cues to navigate the Morris water maze^52^. Given that AD effects egocentric and allocentric navigational strategies, it is possible that navigation using proximal cues contributed at least in part to previously reported deficits. However, we explicitly tested the ability to use distal cues for navigation and found deficits early in disease progression, suggesting that use of distal cues to get oriented is dysfunctional in mouse models of amyloidosis. This failure to use distal cues suggests that future research should explore the possibility that such a deficit may explain the early dysfunction in navigating surroundings in humans with preclinical AD^7^.

Interestingly, differences between males and females were only significant in the dorsal, but not ventral hippocampus, and the dorsal region is linked to performance on the spatial reorientation task and was disassociated from ventral hippocampus on other spatial navigation tasks^34,44–47^. This suggests a potential neural correlate for the early navigational dysfunction in humans with AD. Amyloid pathology may be producing changes in neural function. For example, intracellular amyloid pathology has been shown to interfere with trafficking to the synapse, producing the synaptic dysfunction which appears very early in disease progression^40^. In addition, Aβ has been shown to impact the formation of new proteins and dendritic spines involved in memory processes^40,53^, for review see^54^. Further, Aβ can interact and impact many neuronal receptors leading to the disruption of neural function^54^ and, in particular, can interfere with glutamatergic receptors, which could then contribute to the recruitment of microglia and potentially lead to microglia-mediated synapse loss^55^. Finally, there are other potential mechanisms for the observed impairments. For example, the lack of differences in pathology in cortical regions does not mean that functional differences in these regions are absent. For example, Khan *et al*.^28^ showed that Tau pathology in entorhinal cortex and hippocampus produce a specific functional deficit in PC, even though pathology in this region was much lower than in hippocampus and entorhinal cortex. Thus, there are likely multiple mechanisms driving the effects we observed.

In humans, AD has been shown to be more prevalent in females, including early stages of disease progression^56^. Similarly, we observed that 6-month 3xTg-AD female, but not male 3xTg-AD mice, were impaired at spatial reorientation. The sex difference in spatial reorientation we reported here is consistent with previous reports showing a similar sex difference in Morris water maze deficits at a similar point in disease progression in 3xTg-AD mice^17,48^. We also observed less pathology in male mice, consistent with the lack of impairment with males. Interestingly, an Aβ ELISA did not detect sex differences in pathology, suggesting the ELISA approach may be less sensitive (compared to measures of Aβ positive cells) for detecting a sex difference in pathology at least at this early point in disease progression^48^. In contrast to the present results for spatial reorientation and previous reports using the water maze task where females performed worse, males were impaired on the Barnes maze spatial navigation task, but females were not^15^. The relationship between the sex difference and brain pathology was not described for that report, so it is unclear if variability in brain pathology or other factors contributed to this contradictory result.

The ability to successfully reorient is correlated with the realignment of the hippocampal place field map from start box centered coordinates to room centered coordinates, and aged rats have both poorer realignment to room centered coordinates and also impaired spatial reorientation^34^. Together, this suggests that a similar dysfunction in hippocampal place field realignment to distal cues may be the cause of the behavioral impairment observed in female 3xTg-AD mice. Mouse models of tauopathy and familial AD have shown changes in hippocampal coding and activity patterns^57,58^. Interestingly, the entorhinal cortex is also dysfunctional in AD models that mimic tauopathy, suggesting a larger brain network might be involved in spatial navigation impairments^59^. Future studies could directly examine the possibility that impaired hippocampal place field map alignment is ultimately responsible for the deficits we observed by recording from the hippocampus of 3xTg-AD mice while performing this task.

Navigation deficits are also a key feature of humans with AD, emerging early in disease progression^5–7^, for review see^51^. The findings reported here offer a potential explanation for impaired navigation early in disease progression. Such an impairment may be a consequence of impaired use of landmarks for getting oriented in space. The task used here requires the mouse to use features of the environment to determine the new starting location, which would be a critical feature of navigating, particularly if an individual was navigating new environments. Interestingly, most human and animal studies have focused on allocentric (map-like) aspects of spatial navigation; however, a recent report showed that a distinguishing feature of human AD is the presence of both egocentric (viewer-dependent) and allocentric navigational impairments^20^. In fact, the spatial reorientation task requires translating between egocentric (viewer dependent) information about the landmarks and allocentric (map-like) representations of space^21–23,27,31,43,60–62^. This suggests that hippocampal and even entorhinal cortex changes may not be sufficient to explain the navigational deficits we and others have observed, and that future studies should examine both pathology profile and also function of the hippocampus and a larger brain network for spatial navigation in order to understand the changes underlying navigational impairments in AD.

In summary, we have shown a sex-dependent impairment in spatial reorientation in 3xTg-AD mice early in disease progression. The spatial reorientation task we utilized specifically taxes the use of distal cues to get oriented in space. This use of distal cues to navigate requires translation between viewer dependent and allocentric (map-like) reference frames, thus setting the stage for future studies examining the brain systems that underlie this ability in AD.

## Data Availability

The datasets generated during and/or analyzed during the current study are not publicly available. However, the datasets are available from the corresponding author on reasonable request.
